# Similar Features, Different Behaviors: A Comparative In Vitro Study of the Adipogenic Potential of Stem Cells from Human Follicle, Dental Pulp, and Periodontal Ligament

**DOI:** 10.3390/jpm11080738

**Published:** 2021-07-28

**Authors:** Melissa D. Mercado-Rubio, Erick Pérez-Argueta, Alejandro Zepeda-Pedreguera, Fernando J. Aguilar-Ayala, Ricardo Peñaloza-Cuevas, Angela Kú-González, Rafael A. Rojas-Herrera, Beatriz A. Rodas-Junco, Geovanny I. Nic-Can

**Affiliations:** 1 Facultad de Ingeniería Química, Universidad Autónoma de Yucatán, Periférico Norte Kilómetro 33.5, Tablaje Catastral 13615, Chuburná de Hidalgo Inn, Mérida 97203, Yucatán, Mexico; melissamercadorubio@gmail.com (M.D.M.-R.); erickperezargueta@gmail.com (E.P.-A.); alejandro.zepeda@correo.uady.mx (A.Z.-P.); rafael.rojas@correo.uady.mx (R.A.R.-H.); 2 Laboratorio Translacional de Células Troncales-Facultad de Odontología, Universidad Autónoma de Yucatán, Calle 61-A X Av. Itzaes Costado Sur “Parque de la Paz”, Col. Centro, Mérida 97000, Yucatán, Mexico; faguilar@correo.uady.mx (F.J.A.-A.); pecuevas@correo.uady.mx (R.P.-C.); 3 Unidad de Bioquímica y Biología Molecular de Plantas, Centro de Investigación Científica de Yucatán, Calle 43 No. 130, Col. Chuburná de Hidalgo, Mérida 97200, Yucatán, Mexico; angela@cicy.mx; 4 CONACYT-Facultad de Ingeniería Química, Universidad Autónoma de Yucatán, Periférico Norte Kilómetro 33.5, Tablaje Catastral 13615, Chuburná de Hidalgo Inn, Mérida 97203, Yucatán, Mexico

**Keywords:** adipogenesis, cellular differentiation, dental tissue-derived mesenchymal stem cells, dental pulp cells, dental follicle cells, periodontal ligament cells

## Abstract

Dental tissue-derived mesenchymal stem cells (DT-MSCs) are a promising resource for tissue regeneration due to their multilineage potential. Despite accumulating data regarding the biology and differentiation potential of DT-MSCs, few studies have investigated their adipogenic capacity. In this study, we have investigated the mesenchymal features of dental pulp stem cells (DPSCs), as well as the in vitro effects of different adipogenic media on these cells, and compared them to those of periodontal ligament stem cells (PLSCs) and dental follicle stem cells (DFSCs). DFSC, PLSCs, and DPSCs exhibit similar morphology and proliferation capacity, but they differ in their self-renewal ability and expression of stemness markers (e.g *OCT4* and *c-MYC*). Interestingly, DFSCs and PLSCs exhibited more lipid accumulation than DPSCs when induced to adipogenic differentiation. In addition, the mRNA levels of adipogenic markers (*PPAR*, *LPL*, and *ADIPOQ*) were significantly higher in DFSCs and PLSCs than in DPSCs, which could be related to the differences in the adipogenic commitment in those cells. These findings reveal that the adipogenic capacity differ among DT-MSCs, features that might be advantageous to increasing our understanding about the developmental origins and regulation of adipogenic commitment.

## 1. Introduction

Human mesenchymal stem cells (MSCs) are multipotent cells that can be found in several niches throughout the body, including fat tissue, bone marrow, and brain and dental tissues [1,2,3]. Among these, dental tissue-derived MSCs (DT-MSCs) have received considerable attention due to their self-renewal capacity and immunomodulatory properties. Additionally, DT-MSCs exhibit all positive markers for MSCs, as well as several embryonic stem cell markers, and the notable capacity to differentiate into multiple cell types [4,5].

As a common progenitor of osteocytes and adipocytes, MSCs maintain a delicate balance among chemical, physical, and biological factors that trigger signaling pathways, converging in the tight regulation of transcriptional factors that determine lineage commitment, either for osteogenesis or for adipogenesis [6]. However, the conversion of MSCs to functional adipocytes remains a challenge. Affeldt et al. [7], by using human mesenchymal progenitor cells (MPCs), showed that immediately after overexpressing *PPAR*γ*2*, a key regulator of adipogenesis, the generation of adipocytes reached 90%, ten-fold more than that of non-transduced MPCs. A more recent study highlights that human bone marrow MSCs (BMSCs) have preestablished enhancers, promoting a higher expression of osteoblast-selective genes than adipocyte-selective genes [8]. These findings suggest that the adipogenic pathway could require a wider genetic remodeling program before the proper conversion of MCSs into adipocytes.

Therefore, it would be useful to elucidate the adipocyte development origin to identify mechanisms that block its expansion, since a homeostasis disequilibrium in these cells leads to obesity and obesity-related diseases. This effect means that, aside from the main subcutaneous and visceral fat depots, adipocyte precursors can also have multiple tissue origins, which could be detrimental if they are overdeveloping in tissues such as skeletal muscle or bone marrow [9,10]. In addition, there are many less-studied depots where adipocytes can be generated, and DT-MSCs are among them. In the cranial zone, fat depots contribute to facial shape, and similar to DT-MSCs, a subset of adipocytes originates from neural crest cells [11,12]. During tooth development, the neural crest plays an essential role in the development of the dental mesenchyme which, in turn, gives rise to the dental pulp and follicle, among other important dental cells [13]. Regarding the periodontal ligament (PDL), it is a fibrous network connecting the cementum of the tooth root and the alveolar bone, which preserves the homeostasis of the teeth through their continuous regeneration [14]. Dental pulp, follicle tissue, and periodontal ligaments contain precursor cells called dental pulp stem cells (DPSCs), dental follicle stem cells (DFSCs), and periodontal ligament stem cells (PLSCs), respectively, which can differentiate into many cell types, including adipocytes [4,15,16], the reason for which has been considered as a valuable tool for regenerative treatments and immunotherapies.

Unlike osteogenic differentiation studies, adipogenic differentiation from DT-MSCs has received limited attention. In particular, the differentiation of dental stem cells and others MSC populations into adipocytes has only been used to satisfy the criteria indicated for the International Society for Cellular Therapy [17]. Besides, some studies only show the presence of lipid vacuoles, but the differentiation efficiency is not indicated. Adding to this inconvenience, and despite dental stem cells being induced to adipogenesis, the concentration of the main factor, insulin ((INS), isobutyl-methyl-xanthine (IBMX), dexamethasone (DEXA), and indomethacin (INDO)), varies considerably among different studies [15,18,19], compromising the comparison and reproducibility of the results. Therefore, in this study, we evaluated the in vitro influence of different adipogenic media for their stimulation potential on DPSCs, and then compared the response with DFSCs and PLSCs. To the best of our knowledge, this report is the first to describe the comparative capacity with regard to the adipogenic commitment between three populations of dental stem cells.

## 2. Materials and Methods

The use of the teeth in the present study was approved by the ethics research committee of Dr. Hideyo Noguchi, Regional Research Center, Universidad Autónoma de Yucatán (approval number CIE-06-2017). 

### 2.1. Sample and Cell Culture

Human third molar teeth were obtained from the Clinics of the Master in Pediatric Dentistry and Oral Surgery, Faculty of Dentistry, Autonomous University of Yucatan (UADY), with previously signed written informed consent letters. The dental pulp (DP) was isolated from a two-supernumerary tooth (female, eight years, and male, 12 years), whereas the dental follicle (DF) was separated from the surface of a normal impacted third molar (female, 22 years), and the periodontal ligament (PL) was isolated from a supernumerary tooth (male, 12 years). Cells from all tissues (DF, DP, and PL) were obtained through the outgrowth method. Briefly, all tissues were minced into small pieces (1–2 mm) and placed in 35-mm culture dishes containing alpha-modified Eagle’s medium (α-MEM, Gibco, Grand Island, NY, USA) supplemented with 15% fetal bovine serum (FBS, Gibco, USA) and 1% antibiotic (penicillin/streptomycin, Gibco, Grand Island, NY, USA). The cells were incubated at 37 °C in 5% CO_2_ with saturated humidity and a medium change twice a week. At approximately 80% confluence, the cells were collected after a 3 min digestion with Trypsin-EDTA 0.25% (Gibco, Grand Island, NY, USA) and sub-cultured in a T-25 flask. Morphological characterization of dental cell lines was performed using an inverted phase contrast microscope.

### 2.2. Cell Growth and Viability Assay

The determination of the growth parameters of the cultures derived from DF, DP, and PL was carried out with the objective of knowing the dynamics of cell populations under in vitro conditions. To obtain the proliferation curve of the cells derived from DF, DP, and PL, 1 × 10^4^ cells/mL from cell passage 4 (P4) were seeded in 35 mm petri dishes (two technical replicas per tissue) with 1 mL of α-MEM medium supplemented with 10% FBS and 1% of the antibiotic/antifungal solution, changing the medium every two days. The growth of the in vitro cultures was monitored on days 2, 4, 6, 8, 10, 12, and 14. The cell number and viability were assessed after 14 days by trypsinization (three replicates for each time point), stained with trypan blue (0.4% in PBS 1X) (PBS 1X: 138 mM of NaCl, 3 mM of KCl, 8.1 mM of Na_2_HPO_4_, and 1.5 mM of KH_2_PO_4_, pH = 7.4), and counted using a hemocytometer under a light microscope. Colony-forming assays were plated at a density of 300 cells/dish (35 mm diameter) and cultured in basal medium (α-MEM medium supplemented with 10% FBS) for 14 days under standard culture conditions. All cell cultures were fixed for 10 min with 5 mL of 4% paraformaldehyde (PFA, Sigma-Aldrich, St. Louis, MO, USA) and stained with 0.05% crystal violet (Sigma-Aldrich, St. Louis, MO, USA). The cells were washed twice with distilled water, and the number of colonies was registered. Fifty or more clustered cells were considered colonies.

### 2.3. Immunocytochemical Analysis

To identify stem cell characteristics at P4, DPSCs, DFSCs, and PLSCs were seeded on poly-L-lysine-coated glass cover slips and cultivated for two days (~80% confluence). Cells were fixed with 4% PFA in 1X PBS for 30 min at room temperature (RT) and washed three times with ice-cold 1X PBS. After permeabilization with 0.2% Triton X-100 (Sigma-Aldrich, USA) in 1X PBS for 10 min, the cells were washed with 1X PBS, and nonspecific binding sites were blocked with 3% bovine serum albumin (BSA, Sigma-Aldrich, USA) in 1X PBS for 30 min at RT. After three washes with 1X PBS, cells were incubated with mouse monoclonal antibody against CD105 (Merck-Millipore, Darmstadt, Germany) overnight at 4 °C. Cells were then washed twice with 1X PBS and incubated for 60 min at RT with Alexa Flour^®^ 488 (Invitrogen, Carlsbad, CA, USA). Both primary and secondary antibodies was diluted in a blocking solution at 1:100. Nuclei counterstaining was performed with Vectashield mounting medium (Vector Laboratories, Burlingame, CA, USA), with 4,6-diamidino-2-phenylindole (DAPI) to stain DNA. The immunocytochemical images were acquired with a confocal laser scanning microscope (Olympus, F1V100 SW) and a FV10 ASW 3.1 viewer software. 

### 2.4. In Vitro Differentiation Assays

Osteogenic differentiation: DFSCs, DPSCs, and PLSCs at P4 were seeded at a density of 3 × 10^4^ cells/well in a six-well plate and cultured for 48 h in α-MEM medium supplemented with 10% FBS before osteogenesis or adipogenesis induction. For osteoblast differentiation, cells were cultured in proliferation medium (α-MEM medium + 10% FBS) supplemented with 50 μM of ascorbic acid, 10 mM of *β*-glycerophosphate, and 10 nM of dexamethasone (all purchased from Sigma-Aldrich, USA) for two weeks, as described by Gopinathan et al. [20]. After osteogenic induction, cells were fixed with 4% PFA for 15 min at RT, and mineral formation under differentiation was identified by staining with 2% alizarin red S (Sigma-Aldrich, St. Louis, MO, USA). An inverted microscope (LABOMED) was used to capture digital images.

Adipogenic differentiation: a total of 3 × 10^4^ DFSCs, DPSCs, and PLSCs at P4 were seeded into each well of a six-well plate until 80% confluence. To investigate the differentiation potential of DPSCs towards adipogenesis differentiation, two biological samples of DPSCs were cultured in different adipogenic induction media (AIM, Table 1). Thereafter, the adipocytes generated in each AIM from DPSCs were registered after 21 days of induction. Subsequently, the adipocytes generated in each AIM from DPSCs were registered after 21 days of induction. Then, the best AIM from DPSCs were selected to compare the adipogenic capacity with respect to DFSCs and PLSCs. The cultures were maintained for 21 days, and the induction medium was replaced every three days. Subsequently, cells were fixed in 4% PFA, rinsed twice with 1X PBS, and stained with 0.1% Oil Red O (Sigma-Aldrich, St. Louis, MO, USA) in propanol for 5 min, and the intracellular lipid droplets were evaluated using an inverted phase contrast microscope. The stained area of the cell cultures after Oil Red O staining was quantified in terms of percentage using ImageJ software (National Institute of Health).

### 2.5. Reverse Transcription PCR (RT-PCR) and Quantitative PCR

RT-PCR was performed to measure the levels of mesenchymal stem cells markers (*CD73*, *CD90*, and *CD105*), pluripotency-associated gene expression (*NANOG*, *OCT4*, *KLF4*, *c-MYC*, and *SOX2*), and adipogenic markers (*PPARγ*, *LPL*, and *ADIPOQ*). Total RNA was isolated from dental stem cells (passages 2 and 4) using a Direct-zol RNA kit (Zymo Research) according to the manufacturer’s instructions. For cDNA synthesis, reverse transcription reactions were performed with 1 µg of RNA using the SuperScript First-Strand Synthesis System (Invitrogen, Carlsbad, CA, USA) following the manufacturer’s instructions. MyTaq™ DNA Polymerase (Bioline, UK), 1 μM of each primer, and 50 ng/μL of cDNA in a 25 μL volume was used during PCR with the C1000 Touch Thermal cycler (BIO-RAD, Foster City, CA, USA). 

The PCR products were electrophoresed in a 1.5% agarose gel and stained with ethidium bromide (Sigma-Aldrich, St. Louis, MO, USA). Images were acquired using the Gel Doc Xr+ System (BIO-RAD). Glyceraldehyde 3-phosphate dehydrogenase (*GAPDH*) was used as an internal control; qRT-PCR was performed in triplicate using iTaq Universal SYBR Green Supermix (BIO-RAD, CA, USA) in an Eco Realtime PCR System (Illumina, San Diego, CA, USA) and analyzed using EcoStudy software (Illumina, San Diego, CA, USA). Changes in gene expression were calculated relative to *GAPDH* using the 2^−^^ΔΔ^^CT^ method [21]. The primers used for RT-PCR and qPCR are listed in Supplementary Table S1.

### 2.6. Statistical Analysis

Data are presented as the mean ± standard deviation. Experiments were repeated three times. Significance was analyzed using SigmaPlot software. One-way analysis of variance followed by Tukey’s test was used to determine the significant differences among the groups. 

## 3. Results

### 3.1. Isolation and Biological Features of DT-MSCs

**Figure 1:**
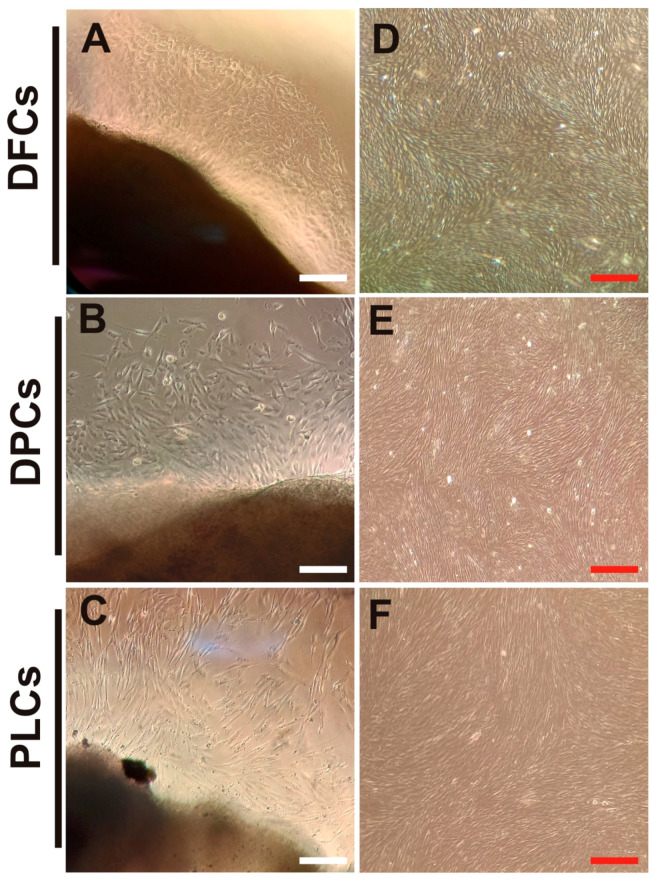
Morphology of primary cultures of human dental follicle cells (DFCs), dental pulp cells (DPCs), and periodontal ligament cells (PLCs). (**A**–**C**) Typical outgrowing fibroblast-like cells from DFCs, DPCs, and PLCs after 10 days of culture. (**D**–**F**) Representative isolated cells at the second passage (P2) of DFCs, DPCs, and PLCs, respectively, showing uniform morphology. The white and red scale bar indicates 150 and 200 µm, respectively.

Primary cultures from human DF, DP, and PL tissues were obtained from a supernumerary tooth and from the surface of a normal impacted third molar, respectively. The outgrowing cells began migrating from explant tissue into the culture dishes between approximately eight and 10 days after seeding. At that time, cell cultures appeared heterogeneous in shape and size (Figure 1A–C). As the cultures progressed, it was observed that the confluence of the DF and PL cells was reached at eight and 10 days, respectively, while, in the DP cells, confluence was not reached until day 12. All primary cells showed a uniform fibroblast-like morphology typical from passage 2 (Figure 1D–F).

**Figure 2:**
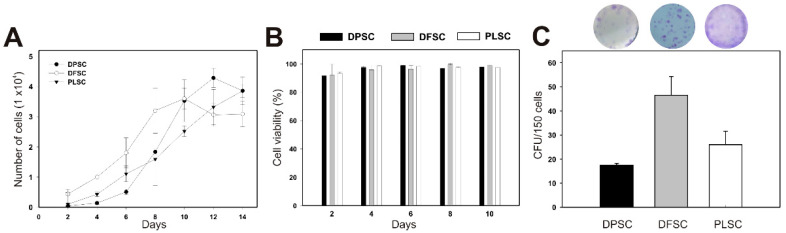
Proliferation, viability, and colony-forming parameters of dental pulp stem cells (DPSCs), dental follicle stem cells (DFSCs), and periodontal ligament stem cells (PLSCs). (**A**) Growth kinetics of DFSCs, DPSCs, and PLSCs cultured in α-MEM + 10% FBS for 14 days at 1 × 10^4^ cells/well. (**B**) Cell viability of dental stem cells (DFSCs, DPSCs, and PLSCs) determined every 48 h for 10 consecutive days. (**C**) Unit-forming colony assay of DFSCs, DPSCs, and PLSCs determined after 14 days of culture. Error bars represent the standard deviation of the mean from three independent experiments (*p* < 0.05).

Our findings indicate that DF, DP, and PL cells at passage 4 exhibited different cell proliferation, as revealed by their corresponding growth curves (Figure 2A). The proliferation rates of DFSCs and PLSCs were slightly higher compared to DPSCs. For instance, DFSCs exhibited a short lag phase after being seeded, reaching a faster plateau phase, while DPSCs were still in the lag phase at day six (Figure 2A), suggesting that these cells could require more time to adapt to the culture conditions. On the other hand, cell viability remained close to 95% on day 10 in all cultures (Figure 2B).

The self-renewal abilities of DFSCs, DPSCs, and PLSCs were determined using the colony-forming assay after 14 days of culture. As shown in Figure 2C, all three cultures analyzed in P4 could form colonies. The DFSCs showed the highest capacity for self-renewal, while DPSCs showed the lowest colony-forming units (CFUs). 

Although colony forming for the three cultures showed similar spindle and fibroblastic morphology, these cell populations exhibited a significant difference in their biological characteristics for colony forming.

### 3.2. Expression of Embryonic and Stem Cell Markers in Dental Stem Cells

**Figure 3:**
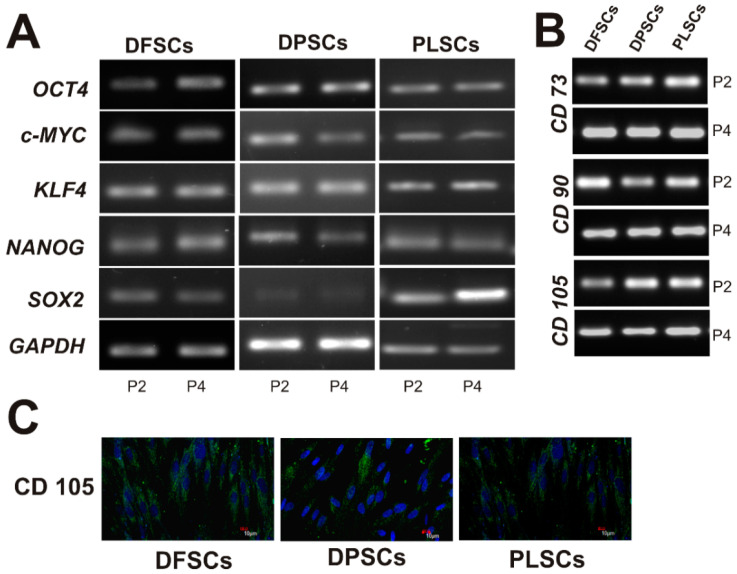
Gene expression profile and immunoflourescence of CD105 of dental stem cells. (**A**) The gene expression profile of embryonic-related genes at passages 2 and 4 (P2, P4) of dental follicle stem cells (DFSCs), dental pulp stem cells (DPSCs), and periodontal ligament stem cells (PLSCs). (**B**) Representative expression profile of mesenchymal stem cell markers (*CD73*, *CD90*, and *CD105*) in DFSCs, DPSCs, and PLSCs at P2 and P4. (**C**) Immunophenotype analysis in DFSCs, DPSCs, and PLSCs by confocal microscopy shows the presence of CD105, a positive mesenchymal marker. Housekeeping gene *GAPDH* was used as an internal control.

To further investigate the stemness properties of DT-MSCs, DFSCs, DPSCs, and PLSCs at P2 and P4 were recollected to evaluate the expression of embryogenic stem cell markers associated with self-renewal and pluripotency, including *OCT-4*, *c-MYC*, *KLF4*, *NANOG*, and *SOX2* by RT-PCR. As shown in Figure 3A, *OCT-4*, *KLF4*, and *NANOG* displayed similar expression levels in all cell lines at P2 and P4. 

Conversely, *c-MYC* expression was slightly more pronounced in the DFSCs at P4 than in DPSCs and PLSCs at the same passage. Interestingly, the expression levels of *SOX2* were variably among all DT-MSCs. For instance, *SOX2* expression was higher in PLSCs, while middle and lower expressions were detected at both passage of DFSCs and DPSCs, respectively. On the other hand, we also verified the expression of mesenchymal stem cell-related markers (*CD73*, *CD90*, and *CD105*) in DFSCs, DPSCs, and PLSCs cultured under basal conditions. The analysis showed that all DT-MSCs exhibited similar expression patterns of these three genes among different dental stem cell populations analyzed, with a homogeneous expression at passage 4 (Figure 3B). In addition, to describe the properties of dental stem cells, adherent cells from DFSCs, DPSCs, and PLSCs were immunostained against CD105, a mesenchymal marker and a membrane glycoprotein involved in the regulation of cell migration. Immunofluorescence showed that all stem cell populations expressed CD105, since DFSCs, DPSCs, and PLSCs exhibited a broad expression of this MSC marker, since ~90% of the cultured cells were positive for CD105 (Figure 3C), consistent with its levels of mRNA (Figure 3B). 

### 3.3. Osteogenic Differentiation Assay

**Figure 4:**
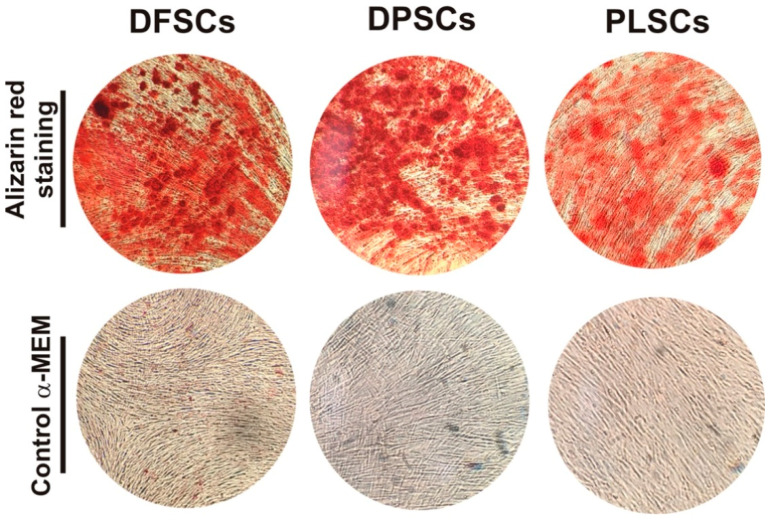
Osteogenic differentiation of DT-MSCs. Osteogenic capacity of dental follicle stem cells (DFSCs), dental pulp stem cells (DPSCs), and periodontal ligament stem cells (PLSCs) determined by calcified mineralized matrix staining with alizarin red after 14 days of induction. No staining was detected in the culture of each dental stem cell in the absence of osteogenic medium.

To determine the plasticity of dental stem cells, DFSCs, DPSCs, and PLSCs were induced to differentiate into osteoblasts for 14 days, and the formation of mineralized nodules was determined by staining with alizarin red. Staining revealed that DPSCs had a higher ability to differentiate into osteoblast than DFSCs and PLSCs, since they were able to form a bundle mineralized nodule indicating great calcium deposits, the area of which was larger than that of DFSCs (Figure 4). Taken together, these results indicated that the cell populations evaluated in this study presented typical mesenchymal stem cell features (Figure 3 and Figure 4).

### 3.4. Effects of Adipogenic Induction Media on Dental Pulp Stem Cells

Although adipogenic differentiation is a basic feature of MSCs, an optimized differentiation method for adipogenesis from dental stem cells is lacking. For instance, the cocktail used to induce adipogenic differentiation from DT-MSCs, including stem cells from human exfoliated deciduous teeth (SHED), PDLSCs, gingival-derived mesenchymal stem cells (GMSCs), tooth germ progenitor cells (TGPCs), DFSCs, and DPSCs, is highly heterogeneous (Supplementary Table S2). Despite these differences, IBMX, INS, DEX, and INDO are the main factors commonly employed for adipogenic induction (Supplementary Table S2). Each cell type has certain particularities in terms of culture media composition, concentration of induction factors, and additional components, and the time induction is also variable [15,18,19].

**Figure 5:**
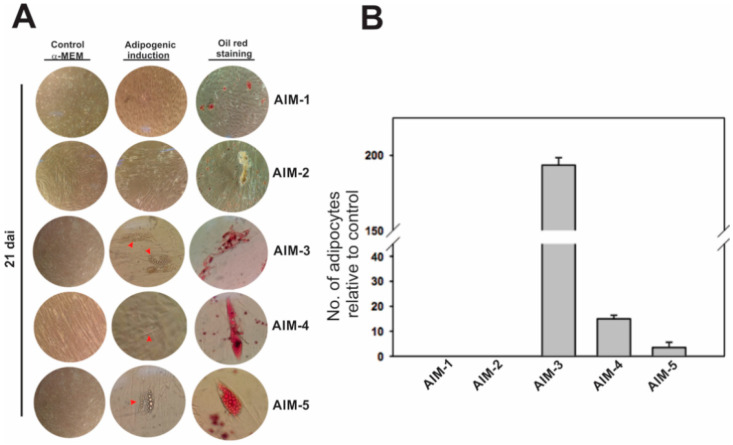
Effects of induction media on the adipogenic capacity of dental pulp stem cells (DPSCs). (**A**) Comparison of different adipogenic induction media (AIM, see Table 1) for induction in DPSCs and Oil Red O staining showing lipid accumulation in adipogenically induced cultures for up to 21 days (21 dai.). Red arrowheads show adipose cells containing lipid droplets. (**B**) Number of adipocytes obtained for each medium shown in A. Values were expressed as means ± SDs of three independent experiments.

Due to existing reports indicating that DPSCs have a limited capacity to differentiate into adipocytes [22,23,24], we first explored the conditions to induce the adipogenic response in those stem cells by comparing them in five adipogenic cocktails (Table 1). As the starting point, we used an adipogenic induction medium 1 (AIM-1, see Table 1), which was enriched with 10 μM of INS and 200 μM of INDO, potent adipogenic agents that stimulate the proliferation and differentiation of preadipocytes by triggering the expression of essential transcriptional factors governing adipogenesis [25,26,27]. However, after 21 days of induction by using AIM-1, we were unable to obtain adipocytes (Figure 5).

Taking into account that, in TGPCs, the use of 1 mM of DEX appears to promote adipogenic induction, even in the absence of INS [28], we thought that an increase in DEX concentration up to ten-fold and a decrease in INS levels (AIM-2) could induce the adipogenic response of DPSCs. Despite these modifications, the differentiation of DPSCs into adipocytes was not possible (Figure 5 and Figure S1). It has been reported that INDO might inhibit adipogenesis in bone marrow stem cells [29]; therefore, we decided to decrease the INDO concentration with the methods reported for SHED [18,30]. It seemed to be that the decreasing INDO concentration from 200 to 60 μM (AIM-3) could be an important step for promoting adipogenesis in DPSCs (Figure 5), since a change in cell morphology and lipid droplet accumulation was detected from the second day and 21 days after induction (dai), respectively (Figure S1). 

DPSCs were able to form adipocytes with lipid droplets (Figure 5), which was confirmed by Oil Red O staining (OROS) 21 dai in AIM-3. Considering that the combination of adipogenic factors of AIM-3 (1.7 μM of INS, 500 μM of IBMX, 1 μM of DEXA, 60 μM of INDO) in α-MEM with 10% FBS led to an adipogenic response, we wondered if the replacement only of the basal culture medium α-MEM by Dulbecco’s modified Eagle’s medium (DMEM/F12, see AIM-4) could improve the differentiation capacity of DPSCs. Although the differentiation of DPSCs into adipocytes was possible using AIM-4 by 21 days, the number of adipocytes were reduced ~13-fold with regard to DPSCs cultured in AIM-3. 

Likewise, by maintaining DMEM/F12 and the same concentrations of induction factors, except INDO, which was increased from 60 to 100 μM (AIM-5), a decreased adipogenic response almost 50-fold was observed when compared to the adipogenic response registered with AIM-3. In this regard, AIM-3 was selected as the medium for the adipogenic induction of DPSCs, and to compare them to DFSCs and PLSCs.

### 3.5. Adipogenic Differentiation of DFSCs, DPSCs, and PLSCs

Although the ability of adipogenic differentiation of DT-MSCs has been reported, the capacity among them is yet to be addressed. Thus, we compared the adipogenic differentiation capabilities of DPSCs relative to DFSCs and PLSCs using AIM-3 medium. The results revealed that these cell populations exhibited a decreased adipogenic capacity in the following order: DFSCs > PLSCs > DPSCs. 

**Figure 6:**
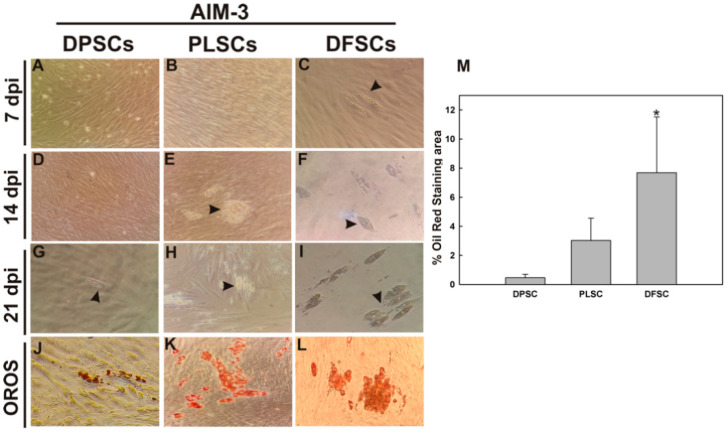
Lipid droplet generation during the adipogenic induction of dental tissue-derived mesenchymal stem cells. Human dental follicle stem cells (DFCSs, **A**,**D**,**G**,**J**), dental pulp stem cells (DPSCs **B**,**E**,**H**,**K**), and periodontal ligament stem cells (PLSCs, **C**,**F**,**I**,**L**) were induced toward adipogenic differentiation by using adipogenic induction medium denominated AIM-3. Black arrowheads indicate the presence of lipid droplets (LDs) detected in the cells at seven and 14 days after induction (dai) in DFSCs (**C**) and PLSCs (**E**), respectively, and at 21 dai in DPSCs (**G**). Adipocyte cells with LDs in red color (**J**–**L**) were visible as indicated by the Oil Red O staining (OROS). (**M**) Quantification of OROS of DFSCs, DPSCs, and PLSCs as a proportion to the Oil Red O positive surface in percentages after 21 days in AIM-3. Error bars represent the standard deviation of the mean from three independent experiments. Asterisks (*) represent significance with respect to the adjacent cell group (*p* < 0.05).

For instance, DFSCs were characterized by a higher adipogenic commitment, since the onset of phenotypic changes was noted as early as the first few days of induction, and the lipid droplets were visible at 7 dai (Figure 6C). In addition, an increased number of adipocytes were easily observable between 14 and 21 dai, as shown by staining cells with OROS 21 dai (Figure 6F,I,L). Important morphological changes were also observed in PLSCs, where the generation of the first cells containing lipid droplets were formed at 14 dai (Figure 6E). Staining revealed a considerable increase in the number of adipocytes differentiated from PLSC cultures after 21 days of culture (Figure 6K).

With respect to adipocyte differentiation in DPSCs, although these population cells exhibited spherical shapes mainly at 14 dai, the first lipids vacuoles were observable in the culture at 21 dai (Figure 6G,J). The ratio of adipogenic responsive cells was confirmed by quantification of OROS from each cell population at 21 days, and the results are summarized in Figure 6M. Consistent with that previously described above, DFSCs and PLSCs exhibited higher stained areas with a peak of 17.4- and 2.43-fold when compared to DPSCs, respectively. Together, these results highlighted that there are important differences in the adipogenic capacity between the different MSC niches of the oral cavity cultured under the same conditions.

### 3.6. Expression Patterns of Adipogenic Markers in Adipocytes Derived from Dental Stem Cells

Adipogenesis is a complex process that requires dramatic changes in the transcriptional program of the cells as a prerequisite to change its shape and its ability to synthesize lipids. In this regard, to determine the expression levels of factors that drive the adipogenic program, the mRNA levels of *PPAR*γ, *LPL*, and *ADIPOQ* were assessed for DPSCs, DFSCs, and PLSCs at 21 dai. PPARγ is considered as a master regulator of adipogenesis, while LPL is involved in lipid transport, and ADIPOQ plays a pivotal role in glucose metabolism and energy homeostasis [31].

**Figure 7:**
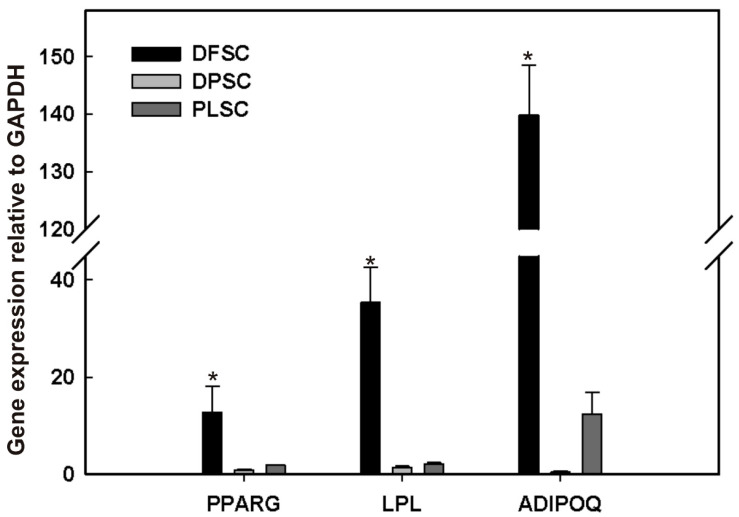
Quantitative PCR analysis of the expression of adipogenic markers *PPARG*, *LPL*, and *ADIPOQ* in cultured dental follicle stem cells (DFSCs), dental pulp stem cells (DPSCs), and periodontal ligament stem cells (PLSCs) under adipogenic conditions after three weeks in the AIM-3 medium (*n* = 3). Error bars represent the standard deviation of the mean from three independent experiments Asterisks (*) represent significance with respect to the adjacent cell group (*p* < 0.05).

As shown in Figure 7, *PPARγ* expression was upregulated in DFSCs > 14- and 6.8-fold than that detected in DPSCs and PLSCs, respectively. *LPL* and *ADIPOQ* were also upregulated in DFSCs > 25- and 283-fold with respect to DPSCs, and 16.6 and 11.23 with regard to PLSCs. These results show that an important difference exists in the expression of adipogenic markers in DPSCs with respect to DFSCs and PLSCs, which seems to be consistent with its poor adipogenic potential (Figure 6).

## 4. Discussion

Although previous studies have shown that DT-MSCs share certain similarities, including morphology, cell surface antigens, expression of stemness markers, and the ability to differentiate into a multitude of cell types, including adipocytes [32,33], there is no study that indicates the reason for their differentiation capacity. In the current study, we compared the mesenchymal features of three lines of DT-MSCs isolated from DP, DF, and PL tissue, and the adipogenic capacity of DPSCs was evaluated by using a custom culture medium. Finally, we compared the adipogenic commitment of DPSCs with that of DFSCs and PLSCs. 

The results revealed that these cell populations differ in certain features at the beginning of the established primary cultures, however, they were similar and indistinguishable (typically elongated fibroblast-like cells) at the second passage (Figure 1D–F) and displayed strong proliferation and viability (Figure 2), which is consistent with previous reports [15,34]. In addition, the isolated DPSCs, DFSCs, and PLSCs showed typical properties of MSCs, since these exhibits similar profile expression of mesenchymal markers (Figure 3B), supporting the presence of stem cell populations [35,36,37] with higher osteogenic potential (Figure 4), comparable to that described elsewhere [20,24,38,39]. In contrast, although DPSCs, DFSCs, and PLSCs contain cell populations with stemness and mesenchymal features, which become evident due to their self-renewal and clonogenic capacity [40,41,42], it is worth noting that differentiation capacity might vary according to their cell origin. 

The fact that DFSCs show higher clonogenicity than DPSCs and PLSCs, for instance, can be explained in part due to DFSCs ontogenic characteristics, since they contain the precursor cells required to give rise to the periodontal tissue attachment apparatus, including alveolar bone, periodontal ligament, and cementum in vivo [33,43], whereas the main function of PLSCs is to repair the damaged tissue, including regeneration of lost alveolar bone [44]. The function of DPSCs is to generate odontoblasts, although there is a possibility that these cells can be found in a more quiescent state [45] with respect to DFSCs and PLSCs. In addition, the nuclei of DFSCs are enriched with dense euchromatin regions [24,46] compared to DPSCs [47] and PLSCs [46], suggesting that DFSCs possess higher transcriptional activity, undifferentiated properties, and possibly better cell plasticity than DPSCs and PLSCs. In this regard, it is known that c-MYC and KLF4 promote cellular proliferation, whereas NANOG, OCT4, and SOX2 are essential for pluripotency maintenance, embryogenic stem cell identity [48], and for inhibiting differentiation-specific gene expression [49], which could partially support our statement about the plasticity of DFSCs and PLSCs over DPSCs. Although the expression of stemness markers can vary according to the population analyzed [22,28,36], it is unknown how the concentration of these transcriptional factors determines dental stem cell plasticity. 

In the field of regenerative medicine, an important aspect to explore is the establishment of optimal culture media for the expansion and differentiation of dental stem cells. However, little has been addressed in this regard. Recently, Di Vita et al. [14] compared the growth characteristics of PLSCs and their response to osteogenic differentiation in α-MEM, DMEM, and enriched Ham’s F12 medium. The authors concluded that the response to differentiation is determined by the formulations of the culture media used and by the role of supplements in triggering cell differentiation. Regarding adipogenic capacity, to the best of our knowledge, standardized techniques for DPSCs differentiation are lacking this in particular, because the differentiation of adipocytes has been used to show the trilineage capacity of dental cells to be considered as MSCs. However, the culture conditions can positively or negatively influence the differentiation capacity of these cells. Therefore, the heterogeneity among different factors used for adipogenic induction might compromise the reproducibility of the results. For instance, a study indicated that DPSCs can give rise to adipocytes by using only ascorbic acid 3-phosphate, DEXA, and INDO [34]. 

Other studies have also reported the conversion of DPSCs and SHED (an immature DPSCs) into adipocytes by using high concentrations of INS and INDO (e.g., 2 mM and 200 mM, respectively), together with DEXA and IBMX [15,19]. However, these studies did not report the quality or efficiency of the differentiation program. It has been reported that increased concentrations of DEXA for a prolonged time promote adipogenesis and inhibit osteogenesis [26]. In contrast, the high concentrations of INDO, a cyclooxygenase inhibitor, seems to decrease the adipogenic capacity of human BMSCs by significantly decreasing *PPARγ* expression [29]. Although INDO is frequently employed to induce adipogenesis in rodents, the requirements on human MSCs could not be the same. In addition, the adipogenic response also appears to be controversial, since, under similar conditions (adipogenic culture media and environmental incubations used), DPSCs can or cannot generate adipocytes [15,22].

In the present study, although we were not able to obtain adipocytes by using a high DEXA, INS, or INDO concentration (AIM-1, AIM-2, Figure 5), the decrease from 200 uM to 60 uM of INDO in the AIM-3 was enough to differentiate adipocytes from two lines of DPSCs obtained from different donors (Figure 5 and Figure 6), suggesting that, in DPSCs, INDO could act as an inhibitor in a dose-dependent manner; however, further studies must be conducted to support this statement. Likewise, the glucose content of basal media also leads to a differential adipogenic response of DPSCs. DMEM/F12 contains threefold more glucose than α-MEM, but the latter stimulates a better adipogenic response in DPSCs (see Table 1 (AIM-3, AIM-4), Figure 5). In support of this finding, the adipogenic commitment in umbilical cord MSCs (UC-MSCs) was improved by changing high-glucose DMEM media to DMEM with low glucose [50]. DMEM is frequently used in the majority of publications on human adipose stem cells (ASCs), BMSCs, or UCMSCs, while α-MEM seems to be preferred for adipogenesis induction in DT-MSCs (Supplementary Table S2). Thus, based on these results, we decided to use only AIM-3 medium to compare the adipogenic differentiation of DPSCs, DFSCs, and PLSCs and their differences in terms of adipogenic marker expression. 

Here, we have shown that the lipid droplets obtained were smaller in DPSCs with regard to those of PLSCs and DFSCs (Figure 6J) isolated and cultured under the same conditions. In accordance with our results, it should also be noted that, independently of the induction medium used, the adipogenic response in DPSCs seems to be sparse in comparison to other dental stem cells, including SHED, PDLSCs, and DFSCs [24,34,41,51,52]. Converting MSCs into relevant adult cell types is not an easy task; in fact, the induced pluripotent stem cells and human embryonic stem cells are also less efficient in their adipogenic capacity [7,53,54]. These findings can be explained by the fact that adipogenesis requires a considerable chromatin remodeling to establish the transcriptional adipogenic network [8,55]. For instance, it has been shown that epigenetic marks, including DNA methylation and H3 lysine 9 dimethylation (H3K9me2), should be removed from the *PPARγ* locus to modulate the adipogenic program [56,57]. This fact enables us to hypothesize that the transcriptional rate of *PPARγ* is not enough to sustain the expression of adipogenesis-related genes, including *LPL* or *AQIPOQ*, in DPSCs (Figure 7), which are essential for lipid metabolism, the concentration of triglycerides, and the generation of active substances, such as adiponectin [58,59].

Besides, this study, as well as others [22,42,47,60], has shown that DPSCs are less able to differentiate into adipocytes with regard to their osteogenic capacity. DPSCs, DFSCs, and PLSCs exhibit osteogenic potential, partly because they are able to generate odontoblast or periodontal osteoblasts among other mineralized tissues [20,44,61]. However, whether the osteogenic-related transcription factors exert a prominent effect on the adipogenic program merits further research. In this regard, one study demonstrated that low adipogenic potential exhibited by DPSCs is due to the upregulation of the phosphatase and tensin homolog (PTEN), a tumor suppressor that defines lineage commitment. The inhibition of this protein by bpV (pic) or the *PTEN* knockdown induces the expression of *PPARγ* and *LPL*, and increases the adipogenic response of DPSCs by suppressing the osteogenic pathway [60]. More recently, despite the fact that DPSCs exhibit MSCs and stemness features, this cell population is unable to differentiate into adipocytes [22] even by using a commercial culture induction media [23]. The detailed analysis of the transcriptomic data showed that DPSCs, when cultured under adipogenic conditions, express genes that inhibit adipogenic differentiation, including the wingless-type member 10 (*WNT10B*) or MSH-like homeobox (*MSX2*) [23], which can prevent adipogenesis by blocking the expression of *PPARγ* and the *C/EBP* family [6].

In contrast, it has also been shown that the overexpression of *OCT4* and *NANOG* enhances the ability of DPSCs to differentiate into adipocytes [35]; unfortunately, the mechanisms by which that occurs is still unknown. Similar results have also been obtained by overexpressing *OCT4* or *SOX2* in ASCs and UCMSCs [62,63], where these transcriptional factors favor the *PPARγ* expression and adipogenic capacity, whereas the loss of *SOX2* function activates the canonical WNT pathway to favor osteogenesis.

Together, these results might help to explain why DPSCs possess limited adipogenic capacity; however, additional transcriptomic and epigenomic studies of dental stem cells (DPSCs, DFSCs, and PLSCs) at different time points will be needed to elucidate the mechanisms governing the activation and repression of the adipogenic program. Additionally, it will also be interesting to determine whether stemness properties play an essential role in influencing cell identity and plasticity among DT-MSCs.

## 5. Conclusions 

Our results demonstrate that DPSCs, DFSCs, and PLSCs differ in their adipogenic potential, despite sharing some mesenchymal features when compared under similar culture conditions. We believe that these findings raise the possibility of identifying new adipogenic precursor cells and the molecular mechanisms governing their development in DT-MSCs, which could increase their use in cell-based therapy and basic research.

## Figures and Tables

**Table 1:** Differentiation media for adipogenic induction of dental pulp stem cells.

Differentiation Cocktail			Treatments				
Basic Conditions	Culture Medium	α-MEM	α-MEM	α-MEM	DMEM/F12	DMEM/F12	α-MEM or DMEM/F12
	FBS (*v*/*v*)	10%	10%	10%	10%	10%	10%
Basic chemical factors	INS	10 μM	1 μM	1.7 μM	1.7 μM	1.7 μM	-
	IBMX	500 μM	500 μM	500 μM	500 μM	500 μM	-
	DEXA	1 μM	10 μM	1 μM	1 μM	1 μM	-
	INDO	200 μM	200 μM	60 μM	60 μM	100 μM	-
Induction time (days)		21	21	21	21	21	21
Adipogenic induction medium (AIM)		AIM-1	AIM-2	AIM-3	AIM-4	AIM-5	Control

## Data Availability

The data associated with in vitro induction to support the findings of this study, including the results and the methods and materials, are included within the article or the Supplementary Materials.

## Supplementary Materials

The following are available online at https://www.mdpi.com/article/10.3390/jpm11080738/s1. **Figure S1.** Representative images of dental pulp stem cells under adipogenesis induction by using different induction media relative to Table 1. Red arrows indicate detected cells with small lipid droplets, characteristics of adipogenic phenotype after 21 days of induction. **Table S1.** Primers sequences used in the experiment. **Table S2.** Adipogenic cocktails used for dental tissue-derived stem cells and adipose-derived stem cells.

## Abbreviations

The following abbreviations are used in this manuscript:

ASCs	adipose stem cells
AIM	adipogenic induction media
α-MEM	alpha-modified Eagle’s medium
BMSCs	bone marrow mesenchymal stem cells
BSA	bovine serum albumin
CFUs	colony-forming units
DAI	days after induction
DAPI	4,6-diamidino-2-phenylindole
DEXA	Dexamethasone
DF	dental follicle
DFSCs	dental follicle stem cells
DP	dental pulp
DPSCs	dental pulp stem cells
DT-MSCs	dental tissue-derived mesenchymal stem cells
DMEM/F12	Dulbecco’s modified Eagle’s medium
FBS	fetal bovine serum
GAPDH	glyceraldehyde 3-phosphate dehydrogenase
GMSCs	gingival-derived mesenchymal stem cells
IBMX	isobutyl-methyl-xanthine
INDO	indomethacin
INS	insulin
LDs	lipid droplets
MPCs	human mesenchymal progenitor cells
MSCs	human mesenchymal stem cells
*MSX2*	MSH-like homeobox
OROS	Oil Red O staining
PBS	phosphate buffer saline
PFA	paraformaldehyde
PL	periodontal ligament
PLSCs	periodontal ligament stem cells
PTEN	phosphatase and tensin homolog
RT	room temperature
SHED	human exfoliated deciduous teeth
TGPCs	tooth germ progenitor cells
UC-MSCs	umbilical cord MSCs
*WNT10B*	wingless-type member 10
