# Supplementary material for: Similar Features, Different Behaviors: A Comparative In Vitro Study of the Adipogenic Potential of Stem Cells from Human Follicle, Dental Pulp, and Periodontal Ligament
Source: J Pers Med. 2021 Jul 28;11(8):738. doi: 10.3390/jpm11080738 (PMC8401480; doi:10.3390/jpm11080738)
Supplement: Supplementary file 1 [file jpm-11-00738-s001.zip › Supplementary materials/S Table S1.pdf]

Supplementary Table S1. Primers sequences used in the experiment.

Amplicon name	Primer sequence (5´-3´)	Used for	References
NANOG	F 5´-TGCTGAGATGCCTCACACGGA-3´<br>R 5´-TGACCGGGACCTTGTCTTCCTT-3´	RT-PCR	1
c-MYC	F 5´-GGACCCGCTTCTCTGAAAGG-3´<br>R 5´- TAACGTTGAGGGGCATCGTC-3´	RT-PCR	1
OCT4	F5´-GAAAGGGACCGAGGAGTA-3´<br>R 5´-CCGAGTGTGGTTCTGTAAC-3´	RT-PCR	2
SOX2	F5´-ACACCAATCCCATCCACACT-3´<br>R 5´-CCTCCCCAGGTTTTCTCTGT-3´	RT-PCR	1
KLF4	F 5´-TACCAAGAGCTCATGCCACC-3´<br>R 5´- CGCGTAATCACAAGTGTGGG-3´	RT-PCR	1
CD73	F 5´-CAGCATTCCTGAAGATCCAAG-3´<br>R 5´- GATTGAGAGGAGCCATCCAG-3´	RT-PCR	3
CD90	F 5´-GTCCTCTACTTATCCGCCTTC-3´<br>R 5´- GACCAGTTTGTCTCTGAGCAC-3´	RT-PCR	3
CD105	F 5´-CTCAAGACCAGGAAGTCCATA-3´<br>R 5´- GATGAGGAAGGCACCAAAG-3´	RT-PCR	3
PPARγ	F 5´-CAGTGGGGATGCTCATAA-3´<br>R 5´-CTTTTGGCATACTCTGTGAT-3´	qPCR	4
LPL	F 5´-ATGGATGGACGGTGACAGGA-3´<br>R 5´-CCAAGACTGTACCCTAAGAGGTG-3´	qPCR	5
ADIPOQ	F 5´-ATGGTCCTGTGATGCTTTGA-3´<br>R 5´-GTTGAGTGCGTATGTTATTTTT-3´	qPCR	4
GADPH	F 5´-CTTTGGCGTGGAAGGACTC-3´<br>R 5´-GTAGAGGCAGGGATGATGTTCT-3´	RT-PCR/qPCR	6

Supplementary References

1. Irie, N.; Weinberger, L.; Tang, W. W.; Kobayashi, T.; Viukov, S.; Manor, Y. S.; Dietmann, S.; Hanna, J. H.; Surani, M. A., SOX17 is a critical specifier of human primordial germ cell fate. *Cell* **2015,** *160* (1-2), 253-68.
2. Hara, E. S.; Ono, M.; Eguchi, T.; Kubota, S.; Pham, H. T.; Sonoyama, W.; Tajima, S.; Takigawa, M.; Calderwood, S. K.; Kuboki, T., miRNA-720 controls stem cell phenotype, proliferation and differentiation of human dental pulp cells. *PLoS One* **2013,** *8* (12), e83545.
3. Bogdanova, A.; Berzins, U.; Nikulshin, S.; Skrastina, D.; Ezerta, A.; Legzdina, D.; Kozlovska, T., Characterization of human adipose-derived stem cells cultured in autologous serum after subsequent passaging and long term cryopreservation. *J Stem Cells* **2014,** *9* (3), 135-48.
4. Karaoz, E.; Demircan, P. C.; Saglam, O.; Aksoy, A.; Kaymaz, F.; Duruksu, G., Human dental pulp stem cells demonstrate better neural and epithelial stem cell properties than bone marrow-derived mesenchymal stem cells. *Histochem Cell Biol* **2011,** *136* (4), 455-73.
5. Kim, D.; Kim, J.; Hyun, H.; Kim, K.; Roh, S., A nanoscale ridge/groove pattern arrayed surface enhances adipogenic differentiation of human supernumerary tooth-derived dental pulp stem cells in vitro. *Arch Oral Biol* **2014,** *59* (8), 765-74.
6. Liu, Z.; Chen, T.; Sun, W.; Yuan, Z.; Yu, M.; Chen, G.; Guo, W.; Xiao, J.; Tian, W., DNA demethylation rescues the impaired osteogenic differentiation ability of human periodontal ligament stem cells in high glucose. *Sci Rep* **2016,** *6*, 27447.
