# Supplementary material for: Similar Features, Different Behaviors: A Comparative In Vitro Study of the Adipogenic Potential of Stem Cells from Human Follicle, Dental Pulp, and Periodontal Ligament
Source: J Pers Med. 2021 Jul 28;11(8):738. doi: 10.3390/jpm11080738 (PMC8401480; doi:10.3390/jpm11080738)
Supplement: Supplementary file 1 [file jpm-11-00738-s001.zip › Supplementary materials/S Table S2.pdf]

Supplementary Table S2. Adipogenic cocktails used for dental tissue-derived stem cells and adipose derived stem cells

**Dental stem cells**		SHED	SHED	SHED	SHED	SHED	DPSCs	DPSCs	DPSCs	DPSCs	DPSCs
**Basic Conditions**	Basal media	DMEM/F12	Dulbeco	α-MEM	DMEM/F12	DMEM/F12	α-MEM	α-MEM	α-MEM	α-MEM	α-MEM
	FBS	10%	10%	10%	10%	10%	10%	10%	15%	10%	10%
**Basic chemical factors**	DEXA	1 mM	1 μM	-	1 mM	1 mM	1 μM	100 nM	-	0.5 μM	10 nM
	INS	-	10 μM	10 μM	1.72 μM	10 μM	0.172 μM	-	1.72 μM	-	2 mM
	IBMX	500 μM	500 μM	500 μM	500 μM	500 μM	500 μM	-	500 μM	0.5 μM	500 μM
	INDO	60 μM	2000 μM	60 μM	60 μM	200 mM	-	140 mM	50 μM	50 μM	-
**Others**	Hidro C	-	-	0.5 μM	-	-	-	-	0.5 μM	-	-
	Asc ac	-	-	-	-	-	-	-	100 μM	-	-
**Induction time**	Differentia-tion (days)	14	21	42	14	14	56	21	28	28	21
**References**		[1]	[2]	[3]	[4]	[5]	[6]	[7]	[8]	[9]	[10]

ASC: human adipose derived stem cells, Asc ac: Ascorbic acid, DPSCs: dental pulp stem cells, DFSCs: dental follicle stem cells, DEXA: dexamethasone, FBS: fetal bovine serum, GMSCs: gingival-derived mesenchymal stem cells, Hidro C: hydrocortisone, INDO: indomethacin, INS: insulin, IBMX: isobutyl-methyl-xanthine, PDLSCs: periodontal ligament stem cells, SHED: stem cells from human exfoliated deciduous teeth. – not reported or not aplicable.

Suplementary Table 2. Adipogenic cocktails used for dental tissue-derived stem cells and adipose derived stem cells (Continue)

**Dental stem cells**		DPSCs DFSCs	PLSCs	PLSCs	GMSCs	ASCs	ASCs
**Basic Conditions**	Basal media	α-MEM	α-MEM	α-MEM	α-MEM	DMEM/F12	DMEM/F12
	FBS	10%	10%	-	10%	10%	10%
**Basic chemical factors**	DEXA	1 mM	-	100 nM	1 nM	1 μM	1 μM
	INS	-	10 μM	-	-	-	10 μM
	IBMX	-	500 μM	500 μM	500 μM	549 μM	500 μM
	INDO	60 mM	60 mM	50 mM	100 μM	66 μM	200 μM
**Others**	Hidro C	-	500 μM	-	-	549 μM	-
	Asc ac	-	100 μM	-	250 μM	-	-
**Induction time**	Differentiation (days)	-	28	14	21	21	21
**References**		[11]	[12]	[13]	[14]	[15]	[16]

ASC: human adipose derived stem cells, Asc ac: Ascorbic acid, DPSCs: dental pulp stem cells, DFSCs: dental follicle stem cells, DEXA: dexamethasone, FBS: fetal bovine serum, GMSCs: gingival-derived mesenchymal stem cells, Hidro C: hydrocortisone, INDO: indomethacin, INS: insulin, IBMX: isobutyl-methyl-xanthine, PDLSCs: periodontal ligament stem cells, SHED: stem cells from human exfoliated deciduous teeth. – no included or reported.

Supplementary References

1. Yalvac, M.E.; Ramazanoglu, M.; Rizvanov, A.A.; Sahin, F.; Bayrak, O.F.; Salli, U.; Palotas, A.; Kose, G.T. Isolation and characterization of stem cells derived from human third molar tooth germs of young adults: implications in neo-vascularization, osteo-, adipo- and neurogenesis. *Pharmacogenomics J* **2010**, *10*, 105-113.

2. Pisciotta, A.; Riccio, M.; Carnevale, G.; Beretti, F.; Gibellini, L.; Maraldi, T.; Cavallini, G.M.; Ferrari, A.; Bruzzesi, G.; De Pol, A. Human serum promotes osteogenic differentiation of human dental pulp stem cells in vitro and in vivo. *PLoS One* **2012**, *7*, e50542.

3. Ma, L.; Makino, Y.; Yamaza, H.; Akiyama, K.; Hoshino, Y.; Song, G.; Kukita, T.; Nonaka, K.; Shi, S.; Yamaza, T. Cryopreserved dental pulp tissues of exfoliated deciduous teeth is a feasible stem cell resource for regenerative medicine. *PLoS One* **2012**, *7*, e51777.

4. Zhang, N.; Chen, B.; Wang, W.; Chen, C.; Kang, J.; Deng, S.Q.; Zhang, B.; Liu, S.; Han, F. Isolation, characterization and multi-lineage differentiation of stem cells from human exfoliated deciduous teeth. *Mol Med Rep* **2016**, *14*, 95-102.

5. Gazarian, K.G.; Ramirez-Garcia, L.R. Human Deciduous Teeth Stem Cells (SHED) Display Neural Crest Signature Characters. *PLoS One* **2017**, *12*, e0170321.

6. Al-Habib, M.; Yu, Z.; Huang, G.T. Small molecules affect human dental pulp stem cell properties via multiple signaling pathways. *Stem Cells Dev* **2013**, *22*, 2402-2413.

7. Navabazam, A.R.; Sadeghian Nodoshan, F.; Sheikhha, M.H.; Miresmaeili, S.M.; Soleimani, M.; Fesahat, F. Characterization of mesenchymal stem cells from human dental pulp, preapical follicle and periodontal ligament. *Iran J Reprod Med* **2013**, *11*, 235-242.

8. Hsieh, S.C.; Tsao, J.T.; Lew, W.Z.; Chan, Y.H.; Lee, L.W.; Lin, C.T.; Huang, Y.K.; Huang, H.M. Static magnetic field attenuates lipopolysaccharide-induced inflammation in pulp cells by affecting cell membrane stability. *ScientificWorldJournal* **2015**, *2015*, 492683.

9. Ponnaiyan, D.; Jegadeesan, V. Comparison of phenotype and differentiation marker gene expression profiles in human dental pulp and bone marrow mesenchymal stem cells. *Eur J Dent* **2014**, *8*, 307-313.

10. Zhang, X.; Li, H.; Sun, J.; Luo, X.; Yang, H.; Xie, L.; Yang, B.; Guo, W.; Tian, W. Cell-derived micro-environment helps dental pulp stem cells promote dental pulp regeneration. *Cell Prolif* **2017**, *50*.

11. Karamzadeh, R.; Baghaban Eslaminejad, M.; Sharifi-Zarchi, A. Comparative In Vitro Evaluation of Human Dental Pulp and Follicle Stem Cell Commitment. *Cell J* **2017**, *18*, 609-618.

12. Hamano, S.; Tomokiyo, A.; Hasegawa, D.; Yoshida, S.; Sugii, H.; Mitarai, H.; Fujino, S.; Wada, N.; Maeda, H. Extracellular Matrix from Periodontal Ligament Cells Could Induce the Differentiation of Induced Pluripotent Stem Cells to Periodontal Ligament Stem Cell-Like Cells. *Stem Cells Dev* **2018**, *27*, 100-111.

13. Zhang, J.; An, Y.; Gao, L.N.; Zhang, Y.J.; Jin, Y.; Chen, F.M. The effect of aging on the pluripotential capacity and regenerative potential of human periodontal ligament stem cells. *Biomaterials* **2012**, *33*, 6974-6986.

14. Van Pham, P.; Tran, N.Y.; Phan, N.L.; Vu, N.B.; Phan, N.K. Vitamin C stimulates human gingival stem cell proliferation and expression of pluripotent markers. *In Vitro Cell Dev Biol Anim* **2016**, *52*, 218-227.

15. Khan, A.; Dellago, H.; Terlecki-Zaniewicz, L.; Karbiener, M.; Weilner, S.; Hildner, F.; Steininger, V.; Gabriel, C.; Muck, C.; Jansen-Durr, P., et al. SNEV(hPrp19/hPso4) Regulates Adipogenesis of Human Adipose Stromal Cells. *Stem Cell Reports* **2017**, *8*, 21-29.

16. Nuermaimaiti, N.; Liu, J.; Liang, X.; Jiao, Y.; Zhang, D.; Liu, L.; Meng, X.; Guan, Y. Effect of lncRNA HOXA11-AS1 on adipocyte differentiation in human adipose-derived stem cells. *Biochem Biophys Res Commun* **2018**, *495*, 1878-1884,
