# Supplementary figures and images for: Similar Features, Different Behaviors: A Comparative In Vitro Study of the Adipogenic Potential of Stem Cells from Human Follicle, Dental Pulp, and Periodontal Ligament
Source: J Pers Med. 2021 Jul 28;11(8):738. doi: 10.3390/jpm11080738 (PMC8401480; doi:10.3390/jpm11080738)

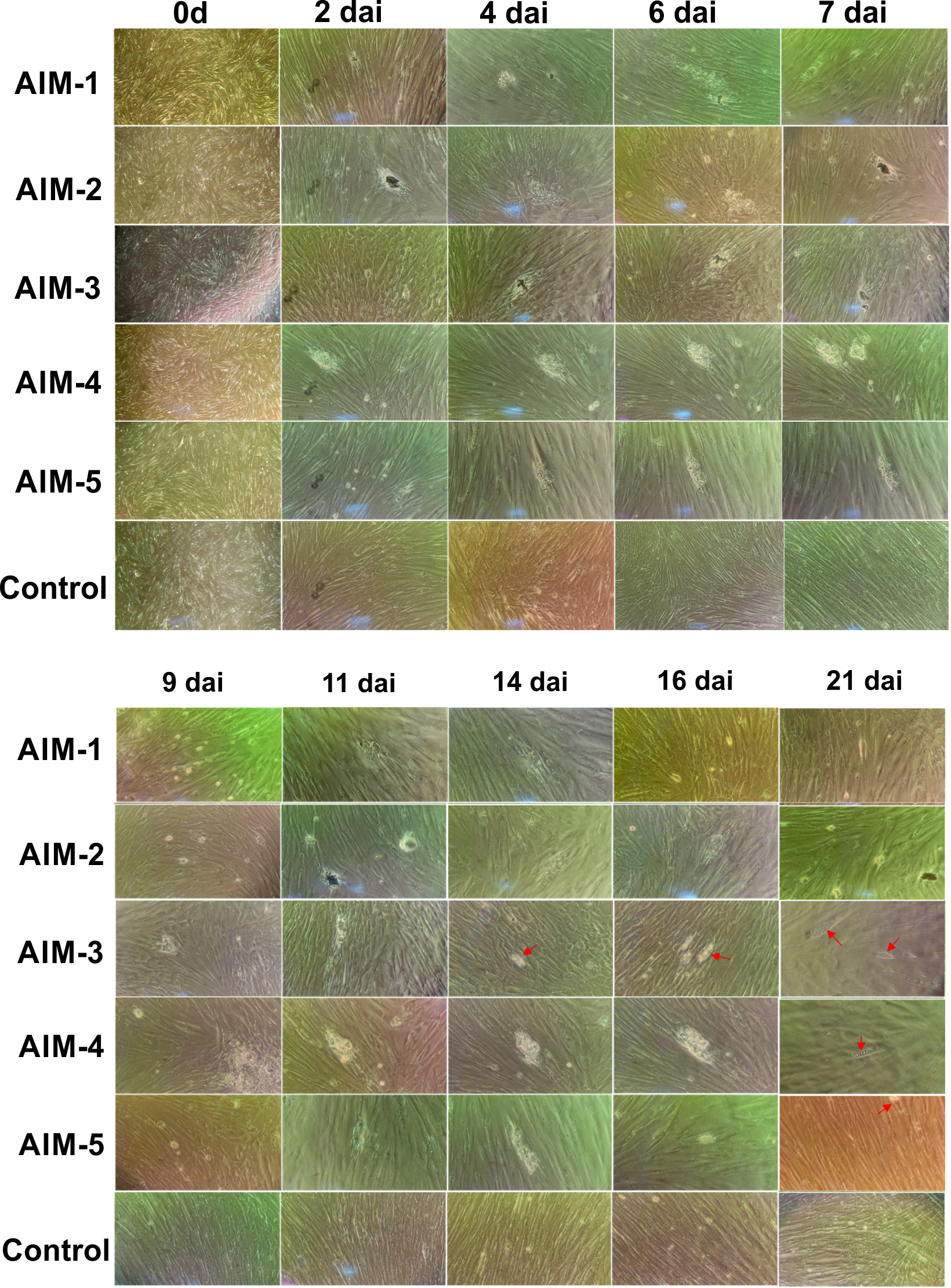

Supplement: Supplementary file 1 [file jpm-11-00738-s001.zip › Supplementary materials/Supplementary Figure 1.TIF]
